# Methodological Insights on Biomarker‐Based Patient Selection: A Review of Scientific Advice Procedures at the European Medicines Agency

**DOI:** 10.1002/cpt.3558

**Published:** 2025-01-18

**Authors:** Cynthia Huber, Joerg Zinserling, Norbert Benda, Thorsten Vetter, Marcia Rueckbeil

**Affiliations:** ^1^ Department of Medical Statistics University Medical Center Göttingen Germany; ^2^ Federal Institute for Drugs and Medical Devices (BfArM) Bonn Germany; ^3^ Scientific Advice Office European Medicines Agency Amsterdam The Netherlands; ^4^ Data Analytics and Methods Task Force European Medicines Agency Amsterdam The Netherlands; ^5^ Department of Medical Statistics University Hospital Aachen Aachen Germany; ^6^ Present address: AbbVie Deutschland GmbH & Co. KG Ludwigshafen Germany; ^7^ Present address: Sanofi‐Aventis Deutschland GmbH Frankfurt am Main Germany

## Abstract

Biomarkers play a pivotal role in the selection and enrollment of trial participants. Particularly, predictive biomarkers help tailor medical care to individual patients; however, also prognostic biomarkers require consideration at the design stage. At the time of initiating a clinical trial, there may be uncertainty about whether a biomarker is predictive or prognostic, and the trial design may need to account for this. Relevant discussions between drug developers and regulators on the role of a biomarker in a specific drug development program are expected to take place during Scientific Advice (SA) procedures. SA procedures at the European Medicines Agency from January 1, 2018, to December 31, 2020, were systematically searched for methodological discussions around the use of predictive or prognostic biomarkers. The final analysis included 45 SA procedures for which key characteristics were summarized quantitatively. Selected methodological issues such as the cutoff selection of continuous biomarkers or study design considerations were elaborated in a qualitative summary. Our results identify commonly encountered points for discussion between drug developers and the European Medicines Agency for biomarker‐informed patient selection and enrollment. Identified topics addressed during SA procedures include cutoff selection, study design, multiplicity control, and data‐driven subgroup selection. The majority of the identified 45 SA procedures concern development programs in oncology. Addressing these issues upfront will allow for an improved dialogue between drug developers and regulators and support the drug development program and ultimately patient‐centered access to medicines.

**Study Highlights**

**WHAT IS THE CURRENT KNOWLEDGE ON THE TOPIC?**

Biomarkers play a crucial role in patient selection and enrollment of trial participants. At the time of initiating a clinical trial, there is often uncertainty about whether a biomarker is predictive or prognostic, and this ambiguity should be accounted for in the trial design. Currently, there is no dedicated regulatory guidance from EMA on biomarker‐based patient selection. However, drug developers can seek advice from EMA on their proposed drug development program, including biomarker‐based patient selection, through Scientific Advice procedures.

**WHAT QUESTION DID THIS STUDY ADDRESS?**

This review aimed to identify commonly encountered methodological points for discussion on biomarker‐based patient selection between drug developers and EMA during Scientific Advice procedures.

**WHAT DOES THIS STUDY ADD TO OUR KNOWLEDGE?**

This review provides an overview of methodological considerations related to predictive and prognostic biomarkers discussed during Scientific Advice procedures between EMA and drug developers. A quantitative summary of key characteristics of the development programs characterizes areas where biomarker‐based patient selection plays an important role. A qualitative description highlights findings from the Scientific Advice procedures on selected methodological topics, such as the cutoff selection for continuous biomarkers and study design considerations.

**HOW MIGHT THIS CHANGE CLINICAL PHARMACOLOGY OR TRANSLATIONAL SCIENCE?**

The findings of this review highlight common points of discussion on biomarker‐based patient selection. Practical recommendations are given which can inform and improve future dialogues between drug developers and regulators on the topic.

Principles guiding medicines development are constantly evolving due to technological and scientific progress. Research developments over the past years have paved the way toward a more patient‐centered approach to medicines development, coining the term of “personalized medicines”, which is described in by the European Union law as “a medical model using characterisation of individuals' phenotypes and genotypes (e.g. molecular profiling, medical imaging, lifestyle data) for tailoring the right therapeutic strategy for the right person at the right time, and/or to determine the predisposition to disease and/or to deliver timely and targeted prevention”.^1^ The terms precision medicine, stratified medicine, and personalized medicine are often used interchangeably.

Biomarkers are defined characteristics that are “measured as an indicator of normal biological processes, pathogenic processes, or responses to an exposure or intervention, including therapeutic interventions”.^2^ If a biomarker carries information about the patient's response to a treatment, this may hence contribute to the development of patient‐centered medical care. Over the last years, there has been an increase in the number of products for which biomarkers form an essential component of medicinal product development and patient selection.^3^ Accordingly, supporting the developments in precision medicine, biomarkers, and omics^4^ was identified as one of the strategic core recommendations in the Regulatory Science to 2025 Strategy by the European Medicines Agency (EMA).^5^ Similarly, the Food and Drug Administration (FDA) has published a draft guidance on Biomarker Qualification in 2018.^6^

Biomarker‐based patient selection in the context of drug development entails methodological challenges linked to the design and analysis of clinical trials. For example, depending on prior knowledge and the (assumed) properties of the biomarker, the trial population may be restricted to a biomarker‐defined population or comprise several biomarker populations to investigate differential biomarker‐based responses to treatment. A review about clinical trial designs used in the context of personalized medicine is given by Superchi et al.^7^ and by Renfro et al.^8^ Ou et al.^9^ discuss best practices and potential issues in biomarker discovery and validation including study designs for validation. Sollfrank et al.^10^ reviewed statistical methods used in studies to investigate biomarker‐related treatment effect heterogeneity in breast cancer patients. They identify two common approaches: the separate estimation of the treatment effect within the biomarker subgroup, and the testing of an interaction term between the biomarker and treatment using regression analysis. Sollfrank et al. notice that studies are often not powered to detect differential treatment effects by biomarker subgroup.

In our review, we sought to identify common methodological challenges in the development and regulatory assessment of biomarkers used for patient selection (trial or target population). In particular, the concepts and aspects of predictive and prognostic biomarkers are of relevance. Predictive biomarkers may be used to identify individuals who are more likely to benefit from the medicinal product under investigation; they are hence of crucial importance for defining suitable patient populations for drug development and approval. Prognostic biomarkers may be used to identify patients who are more likely to experience a certain clinical outcome, regardless of treatment; at the trial design stage, they may be used for enrichment of the trial population,^11^ or as stratification factors.^12^

The term biomarker assay is used to refer to the tool or test used to measure the biomarker. Depending on the measurement outcome of the biomarker assay, patients are often classified as biomarker positive/negative (e.g., if above or below a certain detection limit or cutoff value). For some medicinal products, the biomarker assay used for measurement will be considered a companion diagnostic as defined in Article 2(7) of the In‐Vitro Diagnostic Medical Devices Regulation (EU) 2017/746.^13^ Companion diagnostics are essential for the safe and effective use of the corresponding medicinal product as they are used to identify patients who are most likely to benefit from the medicinal product or at increased risk of experiencing an adverse reaction. Campell^14^ reviews methodological challenges of companion diagnostics, for example, biomarker development, diagnostic performance, and misclassification.

From a perspective of precision medicine, predictive biomarkers are of highest interest as they enable the tailoring of medical treatment to the individual characteristics of patients. In practice, distinguishing between predictive and prognostic biomarkers can often be challenging,^15^ and the generation of firmer evidence on the biomarker's properties may be a crucial part of the drug development program and the regulatory assessment. Furthermore, biomarkers that have been established as being prognostic may provide good candidates for further investigation regarding their predictive properties for a specific medical treatment.^15^ For this reason, we included both predictive and prognostic biomarkers in our review.

Biomarker‐related scientific information is considered as part of several biomarker‐focused or medicinal product‐related procedures at EMA. When it comes to marketing authorization or post‐authorization applications, there is no stand‐alone submission for the biomarker; instead, these data are incorporated into an authorization procedure. Potential discussions about the acceptability of the biomarker may occur upon request by medicinal product developers or applicants, often before submission through the Scientific Advice (SA) procedure.

Scientific Advice is given by the Committee for Medicinal Products for Human Use (CHMP) upon recommendation of the Scientific Advice Working Party (SAWP).^16^ This advice offers an integrated perspective on the quality, safety, and efficacy relating to the development of a medicinal product. The SA procedure aims to optimize Research and Development, reduce uncertainties in regulatory outcomes, and expedite the approval of marketing authorization applications, thereby facilitating timely access of safe and efficacious medicinal products to patients and users of medicinal products. The SAWP is a multidisciplinary group with expertise in nonclinical safety, pharmacokinetics, methodology and statistics, and therapeutic areas such as cardiology and oncology. It includes representatives from the Committee for Orphan Medicinal Products, the Paediatric Committee, the Committee for Advanced Therapies and Pharmacovigilance Risk Assessment Committee.

The conformity assessment of a companion diagnostic is carried out by notified bodies. However, the approval of the corresponding medicinal product is under the remit of medicines' regulatory authorities. Since 2022, there is a consultation procedure by notified bodies to competent authorities for medicinal products for companion diagnostics, some of which fall under the remit of EMA.^17^, ^18^, ^19^ While this consultation procedure is of relevance to companion diagnostics, it is not considered further in our review as it was established after our search period.

Another biomarker‐focused procedure at EMA is the Innovation Task Force, which provides a forum for early dialogue between EMA and sponsors on innovative aspects in medicine development, including innovation in the context of biomarkers.^20^

The Qualification of Novel Methodologies involves the CHMP providing guidance, based on the recommendation of the SAWP, to support the qualification of innovative development methods for a specific context of use in medicinal product research and development.^21^ The novel methodology can apply to nonclinical or to clinical studies, such as the use of a novel biomarker.

Results from a review of biomarker qualification procedures at EMA,^22^ suggest that companies rarely pursue qualification of biomarkers via the biomarker qualification procedures. Especially for biomarkers linked to a specific medicinal product, the authors hypothesize that companies more frequently refer to SA procedures instead.

Since our primary interest was in methodological discussions at the design stage concerning biomarkers that are (potentially) predictive in relation to a medicinal product, we focused our review on SA procedures and did not consider EMA's other regulatory interaction channels. We illustrate common challenges in a qualitative summary. By identifying and highlighting common problems for questions involving the use of biomarkers in the submitted dossiers, we aim at helping drug developers and sponsors to streamline request for scientific advice regarding biomarker development and foster consistent assessment of the dossiers.

## METHODS

### Search of scientific advice procedures

We identified advice letters discussing biomarkers used for patient selection from two sources: EMA's document repository and an EMA database for SA procedures (EMA Scientific Advice database). Both systems include the complete set of advice letters from EMA SA procedures; however, they differ in their search capabilities. Accordingly, the search in both systems was done to mitigate risks of overlooking relevant SA procedures. In EMA's document repository, a systematic search was performed using the following search terms connected with the logical operator “OR”: “Companion Diagnostics,” “Predictive Biomarker,” “Prognostic Biomarker,” “Predictive Marker,” and “Prognostic Marker.”

We restricted our search to final advice letters from closed SA procedures with a date of final adoption by the CHMP between January 1, 2018, and December 31, 2020.

The EMA Scientific Advice database contains additional structured information that is manually entered by the officer responsible for the SA procedure. The additional fields aim to characterize the scope of the SA procedure, including a dedicated field on biomarkers, which shall be selected if the SA procedure covers biomarker‐related aspects.

### Selection of scientific advice procedures

For our review, we selected advice letters from SA procedures that included scientific discussions, specifically questions and comments in the SA letter on methodological issues related to biomarkers used for patient selection.

SA procedures were eligible if the intended context of use for biomarkers was related to patient selection and if there was a contextually relevant methodological discussion around the biomarker's use. SA procedures featuring search strategy terms solely high‐level under general advice or in the bibliography were excluded. For instance, recommendations from the CHMP, such as “consider further subgroup analyses with known prognostic biomarkers,” were categorized as general advice and excluded for further analyses. Similarly, recommendations that merely suggested including or excluding a biomarker as stratification factor without additional justification were also classified as high‐level advice and not incorporated into the analysis. SA procedures that included more detailed descriptions of biomarker‐based subgroup selection were also excluded, if the CHMP did not provide any feedback (e.g., due to the absence of an explicit question to the CHMP).

In contrast, a context was considered relevant if the sponsor's question refers directly to a biomarker‐related issue or when the sponsor's position and/or the CHMP's response include a thorough discussion regarding biomarker‐related issues. We included all types of trial designs in our selection of SA procedures, that is, irrespective of design elements such as the interventional model or study phase.

The selection of SA procedures according to the inclusion and exclusion criteria was performed by one reviewer. SA procedures that did not clearly meet the inclusion and exclusion criteria were assessed by a second reviewer. Additionally, the suitability for inclusion in the review for these cases was discussed by the two reviewers. The discussions addressed any potential ambiguities or discrepancies in the initial assessment.

### Data extraction and classification

The following information was extracted from the included SA procedures. To provide an overview of important features of the SA procedure while respecting confidentiality of ongoing drug development programs, some of the extracted variables were further categorized. The categorization was carried out by one reviewer. A second reviewer then verified it. In cases of uncertainty, a consensus on the appropriate category was reached through discussion.

- Initial/Follow‐up SA
- Procedure number
- Date of initial submission of the SA
- Date of adoption of the final advice letter by the CHMP
- Sponsor
- Sponsor's proposed therapeutic indication of the medicinal product
- based on the proposed indication, the disease area was mapped to the corresponding ICD‐11 chapter
- in case of Neoplasms as ICD‐11 chapter, the disease area was further classified by ICD‐11 subcategory
- Primary endpoint(s) as suggested by the sponsor
- for each SA, we classified whether the sponsor suggested a single primary endpoint, co‐primary endpoints, or dual endpoints
- each single primary endpoint was categorized as time‐to‐event, continuous, binary, or other; co‐primary and dual endpoints were assigned to the respective shared category
- Primary biomarker
- the biomarker was mapped to one of the following biomarker types based on its measurement method: molecular, radiographic, histologic, digital, or physiologic^23^
- Sponsor's position and question on the primary biomarker
- the sponsor's intention to restrict the trial population to the biomarker‐defined subgroup
- the sponsor's view on the anticipated biomarker category was classified: predictive and prognostic, prognostic, predictive, or not clear
- CHMP responses to the biomarker questions
- the CHMP's view on the anticipated biomarker category was classified: predictive and prognostic, prognostic, predictive, or not clear

Some SA procedures addressed more than one biomarker for patient selection. We restricted data extraction to a single biomarker per SA procedure, which was considered to be linked to the most relevant methodological discussions. For example, if a SA procedure included a question on investigating a biomarker in an exploratory analysis as well as another question on conducting the primary efficacy analysis in a potentially predictive biomarker‐defined subgroup, the latter biomarker was chosen for data extraction. The restriction to a single biomarker was performed by a single reviewer and checked by a second one. In case of dissent, the two reviewers reached an agreement through discussion.

The anticipated biomarker category refers to the sponsor's or the CHMP's view of whether the biomarker is predictive or prognostic and does not necessarily imply that this role of the biomarker has already been demonstrated. This view can be influenced by biological plausibility including the drug's mechanism of action, as well as evidence from previous clinical or pre‐clinical trials and other sources such as scientific publications or expert opinions. We classified the anticipated role as “not clear” if the sponsor or the CHMP did neither explicitly nor implicitly refer to the biomarker as being prognostic or predictive. In case of the CHMP, the categorization “not clear” was also chosen if the CHMP considered the presented evidence by the sponsor as insufficient.

### Analysis

Key characteristics of the included SA procedures such as the date of adoption of the final advice letter and the mapped ICD‐11 disease area were summarized descriptively and reported in the text or via bar charts. The anticipated biomarker category according to the sponsor's and the CHMP's perspective, was visualized in an alluvial plot. All quantitative descriptive analyses were conducted using R version 4.3.1.

Key methodological discussions from the included SA procedures were qualitatively analyzed, incorporating both the sponsor's proposal and the comments from the CHMP. An iterative approach was employed to identify and summarize methodological topics across different SA procedures. First, two authors reviewed all included SA procedures independently, taking notes on the methodological topics discussed. Based on the combined notes, the authors then identified recurring methodological topics (“Qualitative analysis” section). Subsequently, for each identified methodological topic, the corresponding SA procedures were reviewed again by one of the authors to summarize key issues across the SA procedures or to select illustrative examples.

To maintain the confidentiality of ongoing drug development programs, our results do not state the sponsor's name, the biomarker name, the primary endpoint, or the therapeutic indication as proposed by the sponsor.

## RESULTS

### Included scientific advice procedures

**Figure 1:**
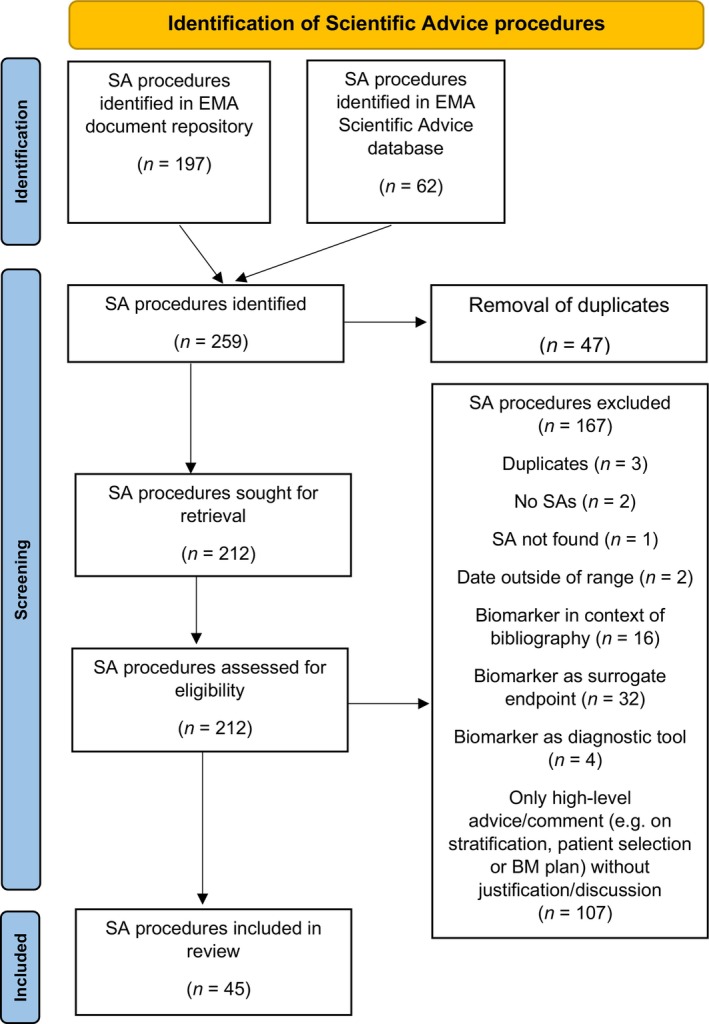
PRISMA Flowchart based on Page et al.^24^

Our search identified 212 distinct SA procedures, which were screened for eligibility. The final analysis included 45 SA procedures, see PRISMA flowchart in **Figure** **1**. A total of 167 SA procedures were excluded (79%). The most common reason for exclusion was that 107 SA procedures included only high‐level biomarker information. In these cases, biomarkers were neither explicitly mentioned in the sponsor's questions nor discussed in detail by the CHMP or the sponsor. Many SA procedures were excluded since the terms biomarker or companion diagnostic only appeared in the bibliography.

### Quantitative analysis

**Figure 2:**
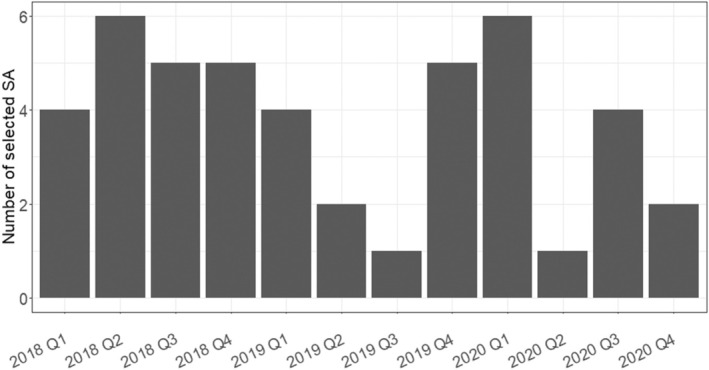
Number of included SA procedures per quarter with dates referring to the date of adoption of the final advice letter by the CHMP.

Data were extracted from the 45 included SA procedures, each focusing on a single main biomarker. Regarding the choice of the main biomarker per SA procedure, only a small number, about five SA procedures, led to a decision regarding a restriction. In two cases, this restriction was less clear due to differences in the established status of the biomarkers. One biomarker was more “established” and used for defining the potential target population but lacked extensive scientific discussion. In contrast, the other biomarker, which we selected for our review, was potentially predictive and prompted more scientific debate between CHMP and the sponsor. **Figure** **2** illustrates the number of included SA procedures per quarter with dates referring to the final adoption date. More than 40% of the 45 included SA procedures were finalized in 2018. In the following years, the number of SA procedures involving relevant discussions on biomarkers decreased. Twelve SA procedures included in our review were finalized in 2019 (27%). In 2020, 13 SA procedures with discussions on biomarkers considered relevant for this review were identified (29%).

Out of the 45 SA procedures included in our review, one was a follow‐up SA procedure whose initial SA is also included in our review. Since the scope of the scientific discussions changed between the initial and the follow‐up SA procedure (e.g., change in biomarker), the follow‐up SA procedure was not excluded from our review.

Among the included SA procedures, a large majority of 36 procedures concerned therapeutic indications in the area of Neoplasms as categorized per ICD‐11 (80%). There were two SA procedures in the area of diseases of the digestive system (4%), two cases in the area of diseases of the immune system (4%), and five cases in other disease areas as per ICD‐11 category (11%). Among the Neoplasms, the most common subtypes were non‐small‐cell lung cancer and breast cancer with nine and seven cases, respectively.

In 38% of the identified SA procedures, sponsors limited the trial population to a biomarker‐defined subgroup, whereas in 60% of the SA procedures, the population was not restricted. Additionally, in one of the identified SA procedures, the sponsor investigated both the biomarker‐restricted and unrestricted populations.

The majority of biomarkers were of molecular nature (69%), followed by histologic biomarkers (22%). Physiologic markers (4%) and radiographic markers (2%) were rare. In one of the SA procedures (2%), it was not possible to categorize the biomarker definitively because the procedure addressed a biomarker development platform for various types of biomarkers.

**Figure 3:**
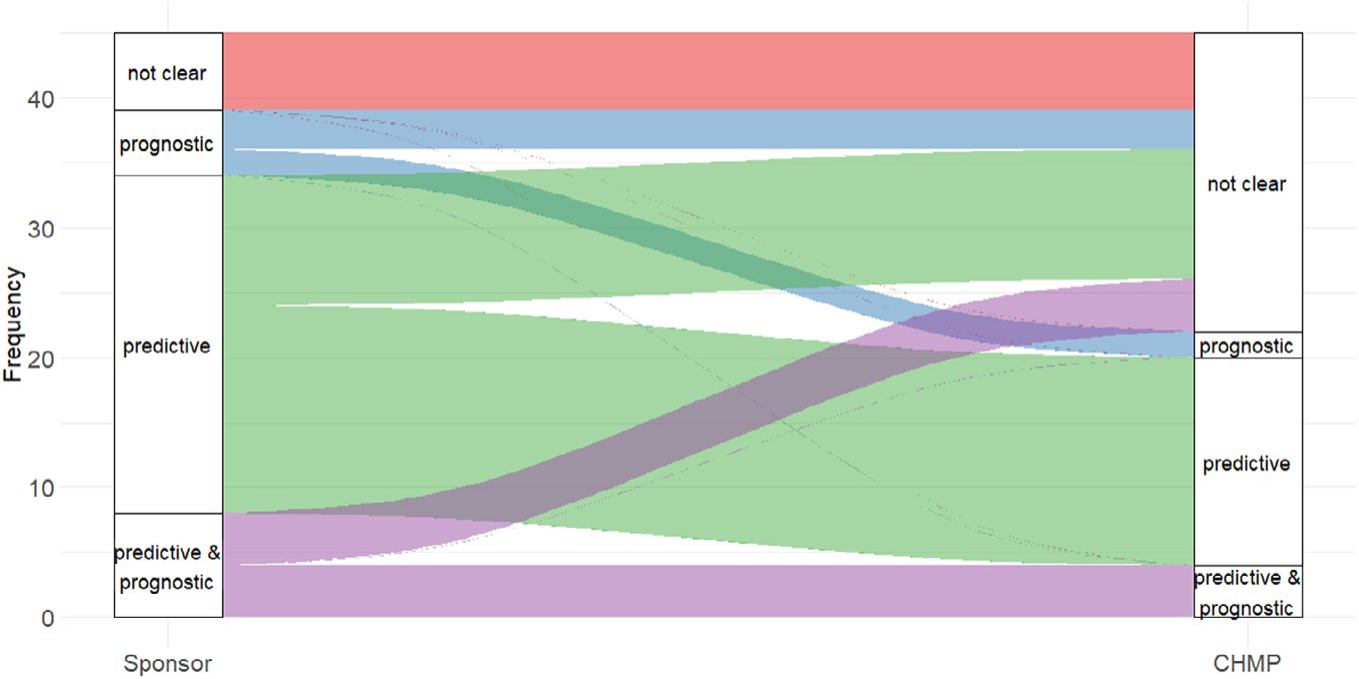
Biomarker category as assumed by the sponsor (left) and by the CHMP (right).

**Figure** **3** shows the interplay between the sponsor's anticipated role of the discussed biomarker (prognostic/predictive) and the corresponding view of the CHMP. As can be seen from **Figure** **3**, sponsors more frequently hypothesized that the biomarker has a prognostic or predictive role whereas the CHMP considered the role as not clear.

The high number of interventions for neoplasms and associated clinical development programs is also reflected in the primary endpoint(s) as suggested by sponsors. The majority of SA procedures (64%) discussed biomarker questions for trials with a time‐to‐event endpoint. In particular, 7% discussed a co‐primary endpoint consisting of progression‐free survival (PFS) and overall survival (OS), while 11% considered a dual primary endpoint of PFS and OS. PFS was the single primary endpoint in 20% of the cases, OS in 13%, and other disease‐state related endpoints in the remaining SA procedures.

### Qualitative analysis

In this section, we summarize biomarker‐related methodological topics that were recurringly discussed in the included SA procedures. Although our summary is supported by illustrative examples, it must be noted that the associated recommendations by the CHMP may be case‐specific and not generalizable to every drug development program.

The scope of SA covers, among others, advice on early and late phase clinical studies with exploratory and confirmatory components. Depending on the targeted context of use for the biomarker‐based patient selection as well as prior knowledge about the biomarker's role, the depth and type of discussion between the CHMP and sponsors varied. For example, for certain oncology indications where PD‐L1 is considered a well‐established prognostic biomarker, sponsors sometimes only provided general literature references, which was deemed sufficient by the CHMP. On the other hand, for novel biomarkers, the CHMP expressed the need for additional compelling evidence about the biomarker's role as supported by data and clinical rationale, for example, factors that influence biomarker levels, biologic plausibility or the drug's mechanism of action. SA procedures on novel biomarkers where the sponsor failed to provide such evidence, were classified as the biomarker having an “unclear” role according to the CHMP (**Figure** **3**).

#### Cutoff selection

The definition of (sub‐)populations based on continuous biomarkers requires the selection of a cutoff value. Methodological considerations in relation to the selection of (sub‐)populations based on a cutoff and the selection of cutoff values are addressed in several EMA guidance documents.^25^, ^26^, ^27^ The cutoff selection was discussed in 11 included SA procedures. In all but two cases, the biomarker was assumed to be predictive according to the sponsor. In the other cases, the biomarker was considered prognostic by the sponsor and planned to be used for enrichment of the study population.

In response to the information as presented by sponsors in their briefing package, the CHMP mainly raised concerns about the sparse information on how the proposed cutoff was selected or the lack of a prospective development plan to identify a suitable cutoff value. For the SA procedures where the sponsor included information on the statistical approaches used to justify a specific cutoff value, these included a variety of visualizations such as boxplots, dot plots, ROC analyses, statistical summary measures such as efficacy and safety results by biomarker subgroup, positive and negative predictive values, sensitivity, specificity and Youden's statistic and other approaches such as a Bayesian logistic regression model. We did not identify SA procedures with detailed feedback by the CHMP on the statistical methodology.

Regarding the CHMP's criticism about insufficient justification for the sponsor's choice of cutoff value, we identified common topics. In a few SA procedures, the CHMP pointed out limitations regarding the examination of a limited range of cutoff values, sometimes advising sponsors to conduct additional (retrospective) analyses to support the cutoff selection. Another source of criticism by the CHMP resulted when sponsors proposed cutoff values from other drug development programs without adequately establishing their relevance to the specific drug development program at hand. For instance, in one SA procedure for a combination therapy, the sponsor presented plans for biomarker‐based patient selection using the same cutoff value for the biomarker assay as had been applied in an early‐phase monotherapy trial. The CHMP requested further justification for the appropriateness of the cutoff value in the combination therapy setting.

Another point that was repeatedly emphasized by the CHMP in response to sponsor's proposals was the requirement to use independent datasets for selection and validation, preferably from two separate studies.

#### Biomarker assays including companion diagnostics

The assay intended for measuring the biomarker was stated in all SA procedures reviewed, with a subset of eight SA procedures including a more detailed discussion on the choice of assay or the measurement methodology. In their advice, the CHMP emphasized the importance of using adequately validated assays^28^, ^29^, ^30^ as well as the different responsibilities by notified bodies vs. the CHMP.^19^ Due to the assumed differential treatment benefit by biomarker levels, it was often recommended to stratify patients according to their biomarker levels.

An important design decision for the biomarker assay is whether to use local or central testing, with the choice depending on the specifics of the drug development program. In one SA procedure, the sponsor proposed a central testing approach using a validated assay. However, the CHMP advised to consider local testing for faster treatment decisions due to known high agreement between central and local testing for the assay in question. In another SA procedure, the sponsor planned to use local testing to determine the eligibility of patients for a clinical trial while simultaneously developing a companion diagnostic. The CHMP stressed the need to re‐test the specimens with the companion diagnostic to confirm concordance with the local test and detailing the approach for handling discordant cases, along with their potential implications on the study results and interpretation.

One sponsor asked about the need to develop a companion diagnostic if the inclusion/exclusion criteria involve biomarkers that are tested as part of standard of care. The CHMP clarified that this would not necessitate the development of a companion diagnostic. However, the CHMP advised to provide clear guidance on the biomarker testing to be used in the clinical trial to limit potential heterogeneity between participating sites.

#### Study design

This subsection concerns methodological biomarker discussions linked to study design components. Questions on the study design were discussed in seven of the SA procedures reviewed.

In a subset of SA procedures reviewed, sponsors and the CHMP disagreed on whether the proposed study design might generate pivotal or exploratory evidence. For instance, in one SA procedure, the sponsor proposed a pivotal uncontrolled two‐stage adaptive phase II trial, with stage I serving to define the biomarker‐based population(s) for stage II. At the end of stage I, results would be analyzed and interpreted by subpopulation as defined by a combination of the disease‐type and biomarker value. A Bayesian testing approach would be used to select the population(s) to advance to stage II. The CHMP noted that stage I might generate exploratory evidence, but, a more thorough justification of the stage II design was required, particularly regarding the intended target population and the rationale for conducting an uncontrolled trial. The CHMP also highlighted the challenges of interpreting data from an uncontrolled trial in a disease with very heterogeneous subpopulations and limited knowledge about whether the biomarker is prognostic or predictive in relation to the investigational treatment.^31^

In one SA procedure, the sponsor proposed an adaptive platform for trials under a shared master protocol with different investigational treatments in pre‐defined (biomarker) subgroups. This platform was meant to identify promising investigational treatments and associated prognostic or predictive biomarkers. The CHMP considered the platform suitable for biomarker identification, pointing out its usefulness to identify prognostic markers based on aggregate data on certain indications. On the other hand, the CHMP highlighted strong concerns about using the platform for validation of predictive biomarkers in relation to an investigational treatment, since such validation requires careful planning and confirmation through independent data.

#### Multiplicity control

Drug approval hinges on identifying a well‐defined target population for which the benefit–risk profile is considered favorable. As such, demonstration of a robust treatment effect within the intended target population involves investigation of the treatment effect in relevant subgroups (internal consistency).^27^ In drug development programs with biomarker‐based patient selection, the investigational treatment is often hypothesized to show a preferable benefit–risk profile with higher efficacy in a specific subgroup. The confirmatory statistical testing strategies are designed accordingly. Among the SA procedures reviewed, five included discussions on multiplicity control in relation to statistical testing in the full trial population vs. a biomarker‐defined subpopulation. Such discussions always referred to biomarkers that were assumed to be predictive for the investigational treatment.

In one SA procedure, the sponsor proposed a hierarchical testing strategy where the primary endpoint would first be tested in a biomarker‐defined subpopulation, followed by testing in the full trial population. In another SA procedure, the sponsor suggested a Bonferroni split, allocating 1% of the alpha to the full trial population and 4% to the biomarker‐defined subgroup. Sample sizes were calculated with the aim to demonstrate efficacy in both populations. In both cases, the CHMP remarked that statistical significance in the full trial population might not be sufficient for approval in the broad target population. Instead, additional analyses in the complementary subgroup would be required to ensure that positive result were not solely driven by the biomarker‐defined subgroup.^27^

#### Data‐driven subgroup selection

The post hoc identification of subgroups with a favorable efficacy or benefit–risk profile after trials show borderline results or fail to establish statistically persuasive evidence in the primary analysis population, pose a serious risk to regulatory approval of the investigational treatment.^27^ In the SA procedures reviewed, two included discussions on data‐driven subgroup selection.

In one SA procedure, the sponsor presented results from a trial where at the trial planning stage, larger values of the biomarker had been assumed to be predictive of superior efficacy. The trial failed its primary efficacy analysis and the sponsor provided post hoc analyses in support of an opposing treatment‐by‐biomarker interaction, that is, with larger values of the biomarker being predictive of inferior efficacy. The CHMP pointed out major methodological limitations and uncertainties in relation to the post‐hoc analyses, emphasizing that such results could only be considered as a basis for hypothesis generation and not be used as confirmatory evidence. In particular, the CHMP expressed major concerns that the observed biomarker data were contrary to the initial clinical hypothesis.

## DISCUSSION

EMA offers the qualification of novel methodologies including biomarkers for a specific context of use (see e.g.,^32^, ^33^). However, this pathway is not frequently pursued in the context of medicinal product specific developments: A recent analysis found that 70% of biomarker‐related Innovation Task Force meetings between 2008 and 2020 did not result in subsequent regulatory qualification or scientific advice interactions, with pharmaceutical companies presumably opting for SA more often.^22^, ^34^ Our search confirmed that the terms predictive and prognostic (bio‐)marker are frequently encountered in SA procedures. However, only in a fraction (45/212) of such advice procedures biomarker‐related issues are explicitly addressed through dedicated questions by sponsors, as we identified with our systematic search. Since the CHMP's responses in SA procedures are given to questions raised by the sponsors, this leads to a much lower proportion of procedures with detailed methodological feedback by the CHMP on biomarker development. Therefore, when seeking scientific advice from EMA regarding biomarker‐based patient selection, it is important to approach the process with careful preparation to obtain valuable feedback. It is necessary to formulate specific questions and provide sufficiently detailed information on the biomarker‐based patient selection, including the chosen study designs, methods, and parameters, such as the selection of cutoff values.

Most of the SA procedures (107/212) were excluded due to high‐level advice or comments on biomarker‐related issues without detailed discussion. Such cases should not collectively be regarded as drug development programs in which biomarkers only play a negligible role. For instance, for drug development programs with very well‐established prognostic (and potentially predictive) biomarkers, the CHMP might recommended to perform additional biomarker‐subgroup analyses without providing a detailed rationale within the SA letter. However, at the time of applying for marketing authorization, the results from this prespecified subgroup investigation could impact the approved indication and thus be of great impact.

The SA procedures included in our review involved diverse biomarker‐related questions and methodological discussions between sponsors and the CHMP. In 2020, a smaller number of finalized SA procedures was identified as compared to the other years. One possible explanation for this might have been the COVID‐19 pandemic; however, the decrease could also be a chance finding. The identified SA procedures pertain to various therapeutic areas but with a vast majority from oncology. We believe that this is primarily because biomarker‐based drug development is very common in oncology and, in part, because oncology is a common therapeutic area for SA procedures.^35^ Since time‐to‐event endpoints are frequently used as primary endpoints in oncology, discussions on biomarkers connected to time‐to‐event endpoints were frequently observed in our review.

One important observation was that in almost half of the SA procedures included, there was a divergence in view between sponsors and the CHMP regarding the anticipated role of the biomarker (prognostic, predictive, or a combination thereof). The divergence mostly arose because the CHMP considered the role of the biomarker to be unclear. This highlights the need for thorough justification and discussion on the presumed role of a biomarker for patient selection in the context of drug development.

Through our qualitative analysis, we identified some methodological topics that were recurrently discussed in the SA procedures related to biomarker‐based patient selection. The definition of topics, their summaries as well as the selection of illustrative examples, involved subjective decision making by the authors. Therefore, an unconscious bias in the definition and selection of topics, as influenced by the authors' respective academic and regulatory backgrounds, cannot be completely ruled out.

One observation from our qualitative analysis was that, according to the CHMP, the choice of cutoff value for the biomarkers was often insufficiently justified or not specific to the drug development program at hand. In addition to available regulatory guidance, several publications cover methodological recommendations on the proper evaluation, interpretation, and validation of cutoff values (see e.g., Polley et al.^36^). Recent research also provides new methods on cutoff selection for biomarkers in clinical studies.^37^, ^38^, ^39^ In terms of study design, discussions focused on the inclusion of comparator arms based on previous results indicating heterogeneous treatment effects in biomarker subgroups, as well as ambiguities regarding the confirmatory nature of the study. The development of novel study designs and analysis methods for investigating differential treatment effects in biomarker‐defined subpopulations has been a research topic of interest for number of years. Ondra et al.^40^ performed a systematic review to identify novel procedures to analyze treatment effect heterogeneity across patient subgroups in clinical trials. They provide an overview of available methodological research and strategies for incorporating biomarkers into the trial design. Apart from one platform trial, and one adaptive two‐stage design, our review identified mostly study designs which can be considered conventional (e.g., parallel group trial, single‐arm trial). However, this is not considered surprising given the time lag and filter of novel trial designs being proposed and the subset of such designs being proposed in regulatory development contexts. For data‐driven subgroup selection, which was also observed in the identified SA procedures, different statistical approaches are available.^41^ However, from a regulatory perspective, data‐driven approaches generally raise major concerns if proposed for confirmatory evidence, especially in trials which fail to meet their predefined success criterion.^42^ Few exceptions may exist, for example, trial designs combining data‐driven subgroup selection within a two‐stage adaptive clinical trial designs have been proposed.^43^ In terms of multiplicity control, we noticed that testing strategies, which prioritize testing in the biomarker‐defined subpopulation over the full trial population (e.g., through hierarchical testing), are not directly covered in EMA's guideline on subgroups.

While not the focus of our review, we observed an inconsistency in terminology for describing the treatment effect in the biomarker‐defined or full trial population across SA procedures. Specifically, from an estimands' perspective, we noticed that instead of clearly characterizing these as changes in the population attribute, sponsors would often describe these as changes in the variable attribute. For example, in the adaptive two‐stage trial from our qualitative analysis, the planned change in population attribute was presented by the sponsor as a change of the endpoint/variable. Given the time frame of our search and the revised version of ICH E9(R1) coming into effect in 2020, this lack of consistency in terminology and approach is not completely surprising. However, it illustrates the benefits of clear scientific communication on the treatment effect of interest, including the use of the estimands' framework in the context of precision medicine.

Biomarker‐based drug development is a complex process which involves several steps. The arguments supporting a biomarker's predictive or prognostic role are therefore usually supported by different sources of evidence, for example, preclinical or early clinical studies, as well as the mechanism of action.^44^ Not every aspect of the biomarker‐based drug development is expected to be discussed with the CHMP. For example, our review did not identify detailed discussions about early, exploratory stages of biomarker discovery.

One limitation of our review was the restriction to SA procedures with a final CHMP opinion between 2018 to 2020. Although we believe that the main methodological insights are still valid, there are some temporal changes that could affect the content of the scientific discussions between sponsors and the CHMP. Examples for potential temporal changes include an improved adoption of the estimands' framework or the entry into force of EU regulation 2017/746 on *in vitro* diagnostics medical devices in 2022,^13^ which has regulatory implications for companion diagnostics.^18^

Another constraint of our search strategy in EMA's document repository is its potential oversight of SA procedures that directly reference biomarkers by name without explicitly using the terms “predictive biomarker,” “prognostic biomarker,” or “companion diagnostics.” This issue is particularly relevant for biomarkers that are widely recognized or routinely studied within specific disease areas, such as PD‐L1 in oncology, where the term “biomarker” may not be explicitly stated in the SA procedure. Moreover, our search strategy is insensitive to different phrasing such as “the biomarker is thought to be predictive.” Furthermore, the terms “predictive” and “prognostic” may not always be used to characterize biomarkers; for example, a treatment‐by‐biomarker interaction might be discussed instead. Additionally, the categorization of the extracted data was performed by a single reviewer. Although a second reviewer verified the categorization and ambiguities were discussed, this approach introduced a level of subjectivity into the quantitative results. Despite these limitations, we consider that the overall risk of missing relevant SA procedures was sufficiently mitigated through the complementary search in the EMA Scientific Advice database. Nevertheless, we recommended using clear and adequate terminology to indicate whether a biomarker is considered predictive or prognostic to avoid ambiguity.

In conclusion, we consider that the search strategy identified a significant number of pertinent SA procedures. The quantitative and qualitative summary offer a good impression of biomarker‐related discussions on patient selection via EMA's Scientific Advice channel. Another important insight from our review concerns the relevant proportion of identified SA procedures for which the sponsor included detailed descriptions on biomarker‐based patient selection without asking explicit questions to the CHMP. Omitting explicit questions bears the considerable risk of not getting a response or only a partial response from the CHMP on the biomarker plans. Similarly, the CHMP will not be able to provide optimal feedback if crucial information such as the statistical analysis plan is missing from the briefing document. In case of biomarker‐based patient selection for drug development, we hence recommend formulating explicit questions on relevant methodological aspects including the provision of adequate justification. The recurring topics for discussion between sponsors and the CHMP (“Qualitative analysis” section) provide an orientation for relevant methodological aspects to consider. We hope that our findings will help to improve the effectiveness of scientific interactions between drug developers and the CHMP.

## FUNDING

No funding was received for this work.

## CONFLICT OF INTEREST

The authors declare no competing interests for this work at the time of conducting the research and preparing the manuscript. However, please note that C.H. has recently transitioned from University Medical Center Göttingen to Abbvie, whereas M.R. has recently transitioned from University Hospital Aachen & EMA to Sanofi‐Aventis. The changes in affiliation occurred after the submission, but before acceptance of this manuscript.

## AUTHOR CONTRIBUTIONS

All authors wrote the manuscript. All authors designed the research. C.H. and M.R. performed the research. C.H. and M.R. analyzed the data.
